# Higher body mass index was associated with better prognosis in diabetic patients with stage II colorectal cancer

**DOI:** 10.1186/s12885-022-09691-1

**Published:** 2022-06-01

**Authors:** Xiao-Yu Liu, Bing Kang, Yu-Xi Cheng, Chao Yuan, Wei Tao, Bin Zhang, Zheng-Qiang Wei, Dong Peng

**Affiliations:** 1 Department of Gastrointestinal Surgery, The First Affiliated Hospital of Chongqing Medical University, Chongqing, 400016 China; 2 Department of Clinical Nutrition, The First Affiliated Hospital of Chongqing Medical University, Chongqing, 400016 China

**Keywords:** Colorectal cancer, Body mass index, Type 2 diabetes mellitus, Prognosis

## Abstract

**Purpose:**

The purpose of this study is to analyze the effect of body mass index (BMI) on patients with concurrent colorectal cancer (CRC) and type 2 diabetes mellitus (T2DM).

**Methods:**

Patients who underwent primary radical CRC surgery from Jan 2011 to Jan 2020 were retrospectively collected. The perioperative information, overall survival (OS) and disease-free survival (DFS) were compared between the higher BMI group and the lower BMI group.

**Results:**

A total of 574 patients with concurrent CRC and T2DM were included in this study. The higher BMI group had higher portion of hypertension (*p* < 0.01) and coronary heart disease (CHD) (*p* < 0.01). Furthermore, the higher BMI group had better OS (*p* = 0.016) and DFS (*p* = 0.040) than the lower BMI group in stage II CRC. In multivariate analysis, age (OS: *p* = 0.002, HR = 2.016, 95% CI = 1.307–3.109/ DFS: *p* = 0.003, HR = 1.847, 95% CI = 1.230–2.772), TNM stage (OS: *p* < 0.01, HR = 1.667, 95% CI = 1.281–2.169/ DFS: *p* = 0.001, HR = 1.545, 95% CI = 1.207–1.977), overall complications (OS: *p* = 0.004, HR = 1.837, 95% CI = 1.218–2.880/ DFS: *p* = 0.006, HR = 1.783, 95% CI = 1.184–2.686) and major complications (OS: *p* = 0.005, HR = 2.819, 95% CI = 1.376–5.774/ DFS: *p* = 0.014, HR = 2.414, 95% CI = 1.196–4.870) were independent factors of OS and DFS. Moreover, BMI (*p* = 0.019, HR = 0.413, 95% CI = 0.197–0.864) was an independent factor of OS in stage II CRC.

**Conclusion:**

Higher BMI was associated with better OS in diabetic patients with stage II CRC.

## Introduction

Colorectal cancer (CRC) is the third most common cancer and the second leading cause of cancer-related deaths worldwide. In 2018, there were nearly 1.8 million new cases of CRC and 881,00 CRC related deaths [1]. The incidence of CRC in China is increasing, especially in economically developed areas [2]. Although there are many treatments for CRC including surgery, radiotherapy, and chemotherapy. Radical surgery is still the cornerstone of the treatment of CRC [3–5].

Type 2 Diabetes mellitus (T2DM) is a metabolic disease characterized by high blood sugar caused by insulin deficiency or resistance [6]. The global burden of T2DM is increasing recently. There are nearly 500 million patients with T2DM worldwide, and it is expected to reach to 629 million by 2045 [7, 8]. T2DM is one of the most common causes of death in the world as well.

Body mass index (BMI) is a commonly used scale for assessing obesity [9]. BMI is not only associated with T2DM, but also has an impact on CRC. Previous studies reported conflicting results about the impact of BMI on CRC. Some studies reported that higher BMI decreased the overall survival (OS) and disease-free survival (DFS) of CRC [10, 11], however, other studies reported that BMI did not affect the prognosis on CRC [12, 13].

Ye Z et al. [14] reported that low preoperative BMI was a poor prognostic marker for T2DM patients with gastric cancer. However, the role of preoperative BMI on prognosis of diabetic patients with CRC was unclear. Therefore, the purpose of this study is to analyze the effect of BMI on patients with concurrent CRC and T2DM.

## Methods

### Study design

This is a retrospective study and the results are reported with consideration to the Strengthening the Reporting of Observational Studies in Epidemiology (STROBE) [15].

### Ethical approval

This study was in accordance with the World Medical Association Declaration of Helsinki. The study was approved by the ethical review board (2021–336), and all patients signed informed consents.

### Patients

We retrospectively collected diabetic patients who underwent primary radical CRC surgery in a single clinical center from Jan 2011 to Jan 2020.

### Inclusion and exclusion criteria

The inclusion criteria were as follows: 1, patients who underwent primary radical CRC surgery; and 2, patients were diagnosed with concurrent CRC and T2DM. According to the inclusion criteria, a total of 702 patients were identified in the current study. The exclusion criteria were as follows: 1, incomplete medical records (*n* = 103); 2, palliative CRC surgery (*n* = 25). Finally, 574 patients with concurrent CRC and T2DM were included in the study.

### Surgery and follow-up

The surgical resection of CRC was according to the clinical guideline. Total mesorectal excision or complete mesocolic excision was performed, and the pathology confirmed R0 resection. Patients were followed up every three months for the first three years and every six months for the following two years. The follow-up items included computed tomography (CT), magnetic resonance imaging (MRI), carcinoembryonic antigen (CEA) or colonoscopy.

### Definitions

Tumor nodes metastasis (TNM) stage was defined according to the AJCC 8^th^Edition [16]. Complications were defined according to the Clavien-Dindo classification [17], and major complications were defined as ≥ III classification complications including patients who needed surgery, endoscopy or interventional operation. OS was defined as the time from surgery to death or last follow-up time. DFS was defined as the time from surgery to recurrence, death or last follow-up time.

### Data collection

The perioperative information was collected from the inpatient system. We collected the perioperative information such as sex, age, BMI, drinking, smoking, coronary heart disease (CHD), family history, tumor location, TNM stage, vessel invasion, perineural invasion, adjuvant therapy, operation time, blood loss, retrieved lymph nodes, overall complications, major complications and hospital stay. The follow-up information including OS and DFS were collected from the outpatient system and telephone interviews.

### Statistical analysis

Patients were divided into two groups according to the median of BMI (the higher BMI group and the lower BMI group), and the cut-off of BMI was 23.4 kg/m^2^. Continuous variables were expressed as the mean ± SD, and independent-sample t test was used to compare the difference between the two groups. Frequency variables were expressed as n (%), and Chi-square test exact test was used. The Kaplan–Meier curve was conducted to compare the difference of BMI on each TNM stage, and cox regression analyses were performed to identify independent predictive factors for OS and DFS. Interaction analyses was conducted in the COX regression model between the baseline information. Data were analyzed using SPSS (version 22.0) statistical software. A bilateral *p* value of < 0.05 was considered statistically significant.

## Results

### Baseline information

**Fig. 1:**
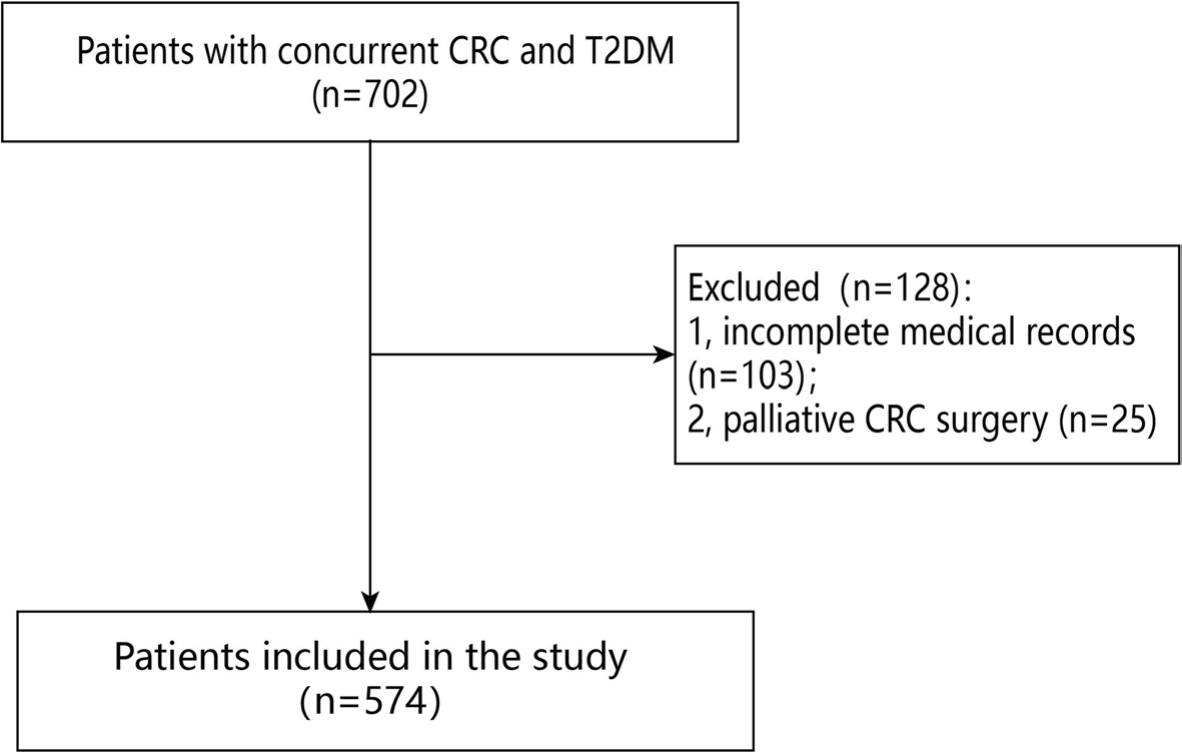
Flow chart of patient selection

A total of 574 patients with concurrent CRC and T2DM were included in this study, and the flow chart of inclusion and exclusion was shown in Fig. 1. There were 336 males and 238 females, and the average BMI was 23.6 ± 3.3 kg/m^2^. The age, tumor site, TNM stage, family history, smoking, drinking, hypertension and CHD were shown in Table 1.

**Table 1:** Baseline characteristics

Characteristics	No.574
Sex	
Male	336 (58.5%)
Female	238 (41.5%)
Age, years	68.2 ± 9.5
BMI, kg/m^2^	23.6 ± 3.3
Smoking	205 (35.7%)
Drinking	172 (30.0%)
Hypertension	319 (55.6%)
CHD	58 (10.1%)
Family history	18 (3.1%)
Adjuvant therapy	39 (6.8%)
Tumor site	
Rectum	288 (50.2%)
Colon	286 (49.8%)
TNM stage	
I	95 (16.6%)
II	233 (40.6%)
III	208 (36.2%)
IV	38 (6.6%)

*Note*: Variables are expressed as the mean ± SD or n (%)

*Abbreviations*: *BMI* Body mass index, *CHD* Coronary heart disease, *TNM* Tumor nodes metastasis

### Comparison between higher BMI group and lower BMI group

The BMI was divided in two groups according to the median, and there were 288 patients in the higher BMI group and 286 patients in the lower BMI group. The perioperative information was compared between the two groups. The higher BMI group had higher portion of hypertension (*p* < 0.01) and CHD (*p* < 0.01), however, there were no difference of other perioperative information (*p* > 0.05) (Table 2).

**Table 2:** Comparison between the higher BMI group and the lower BMI group

Characteristics	Higher BMI (*n* = 288)	Lower BMI (*n* = 286)	*P* value
Age (years)	68.6 ± 9.4	67.8 ± 9.7	0.334
Sex			0.682
Male	171 (59.4%)	165 (57.7%)	
Female	117 (40.6%)	121 (42.3%)	
Smoking	103 (35.8%)	102 (35.7%)	0.980
Drinking	89 (30.9%)	83 (29.0%)	0.443
Hypertension	188 (65.3%)	131 (45.8%)	< 0.01*
CHD	42 (14.6%)	16 (5.6%)	< 0.01*
Family history	12 (4.2%)	6 (2.1%)	0.155
Tumor location			0.933
Rectum	144 (50.0%)	144 (50.3%)	
Colon	144 (50.0%)	142 (49.7%)	
TNM stage			0.249
I	56 (19.4%)	39 (13.6%)	
II	115 (39.9%)	118 (41.3%)	
III	97 (33.7%)	111 (38.8%)	
IV	20 (7.0%)	18 (6.3%)	
Vessel invasion	12 (4.2%)	19 (6.6%)	0.189
Perineural invasion	10 (3.5%)	6 (2.1%)	0.317
Adjuvant therapy	19 (6.6%)	20 (7.0%)	0.851
Operation time (minutes)	232.8 ± 83.9	228.8 ± 85.6	0.577
Blood loss (mL)	105.6 ± 132.0	183.6 ± 10.9	0.568
Retrieved lymph nodes	14.3 ± 10.8	14.9 ± 7.0	0.439
Overall complications	73 (25.3%)	92 (32.2%)	0.071
Major complications	10 (3.5%)	9 (3.1%)	0.828
Hospital stay (days)	12.3 ± 11.9	12.8 ± 10.5	0.562

*Note*: Variables are expressed as the mean ± SD, n (%), *P-value < 0.05

Abbreviations: *BMI* Body mass index, *CHD* Coronary heart disease, *TNM* Tumor nodes metastasis

### Univariate and multivariate analysis of OS/ DFS

The medium follow-up time was 31 (1–113) months. In univariate analysis, age (*p* = 0.001, HR = 1.039, 95% CI = 1.016–1.062), BMI (*p* = 0.049, HR = 0.665, 95% CI = 0.443–0.999), TNM stage (*p* < 0.01, HR = 1.672, 95% CI = 1.294–2.162), vessel invasion (*p* < 0.01, HR = 3.517, 95% CI = 1.760–7.026), overall complications (*p* < 0.01, HR = 2.312, 95% CI = 1.554–3.439) and major complications (*p* < 0.01, HR = 4.398, 95% CI = 2.278–8.493) were significant risk factors. In multivariate analysis, age (*p* = 0.001, HR = 2.089, 95% CI = 1.353–3.227), TNM stage (*p* < 0.01, HR = 1.601, 95% CI = 1.229–2.086), vessel invasion (*p* = 0.006, HR = 2.759, 95% CI = 1.339–5.683), overall complications (*p* = 0.003, HR = 1.936, 95% CI = 1.258–2.980) and major complications (*p* = 0.019, HR = 2.412, 95% CI = 1.157–5.029) were independent factors of OS. Interaction analysis of age and BMI revealed no significant difference (*p* = 0.374 > 0.05) (Table 3).

**Table 3:** Univariate and multivariate analysis of overall survival

Risk factors	Univariate analysis		Multivariate analysis	
	HR (95% CI)	*P* value	HR (95% CI)	*P* value
Age (> / ≤ 68, years)	1.039 (1.016–1.062)	0.001*	2.089 (1.353–3.227)	0.001*
Sex (female/male)	0.798 (0.528–1.207)	0.286		
BMI (> / ≤ 23.4 kg/m^2^)	0.665 (0.443–0.999)	0.049*	0.704 (0.466–1.062)	0.094
Hypertension (yes/no)	0.980 (0.659–1.459)	0.922		
Tumor site (colon/ rectum)	1.396 (0.936–2.048)	0.102		
TNM stage (IV/III/II/I)	1.672 (1.294–2.162)	< 0.01*	1.601 (1.229–2.086)	< 0.01*
Vessel invasion (yes/no)	3.517 (1.760–7.026)	< 0.01*	2.759 (1.339–5.683)	0.006*
Perineural invasion (yes/no)	2.627 (0.823–8.379)	0.103		
Adjuvant therapy (yes/no)	1.024 (0.375–2.797)	0.963		
Smoking (yes/no)	1.202 (0.802–1.803)	0.373		
Drinking (yes/no)	1.334 (0.880–2.022)	0.175		
Family history (yes/no)	1.406 (0.572–3.459)	0.458		
CHD (yes/no)	1.358 (0.742–2.486)	0.321		
Overall complications (yes/no)	2.312 (1.554–3.439)	< 0.01*	1.936 (1.258–2.980)	0.003*
Major complications (yes/no)	4.398 (2.278–8.493)	< 0.01*	2.412 (1.157–5.029)	0.019*

*Note*: *P-value < 0.05

*Abbreviations*: *HR* Hazard ratio, *CI* Confidence interval, *BMI* Body mass index, *CHD* Coronary heart disease, *TNM* tumor nodes metastasis

In terms of DFS, age (*p* = 0.002, HR = 1.876, 95% CI = 1.250–2.814), TNM stage (*p* = 0.001, HR = 1.502, 95% CI = 1.173–1.924), vessel invasion (*p* = 0.010, HR = 2.515, 95% CI = 1.243–5.087), overall complications (*p* = 0.004, HR = 1.834, 95% CI = 1.217–2.765) and major complications (*p* = 0.032, HR = 2.185, 95% CI = 1.070–4.462) were independent factors as well (Table 4).

**Table 4:** Univariate and multivariate analysis of disease-free survival

Risk factors	Univariate analysis		Multivariate analysis	
	HR (95% CI)	*P* value	HR (95% CI)	*P* value
Age (> / ≤ 68, years)	2.039 (1.362–3.053)	0.001*	1.876 (1.250–2.814)	0.002*
Sex (female/male)	0.846 (0.572–1.251)	0.402		
BMI (> / ≤ 23.4 kg/m^2^)	0.721 (0.491–1.059)	0.096		
Hypertension (yes/no)	1.003 (0.686–1.465)	0.989		
Tumor site (colon/ rectum)	1.364 (0.932–1.995)	0.268		
TNM stage (IV/III/II/I)	1.551 (1.217–1.975)	< 0.01*	1.502 (1.173–1.924)	0.001*
Vessel invasion (yes/no)	3.028 (1.521–6.027)	0.002*	2.515 (1.243–5.087)	0.010*
Perineural invasion (yes/no)	2.250 (0.707–7.160)	0.170		
Adjuvant therapy (yes/no)	1.622 (0.750–3.507)	0.219		
Smoking (yes/no)	1.148 (0.779–1.692)	0.485		
Drinking (yes/no)	1.243 (0.833–1.855)	0.287		
Family history (yes/no)	1.316 (0.536–3.230)	0.549		
CHD (yes/no)	1.195 (0.656–2.179)	0.561		
Overall complications (yes/no)	2.123 (1.452–3.104)	< 0.01*	1.834 (1.217–2.765)	0.004*
Major complications (yes/no)	3.981 (2.070–7.657)	< 0.01*	2.185 (1.070–4.462)	0.032*

*Note*: *P-value < 0.05

*Abbreviations*: *HR* Hazard ratio, *CI* Confidence interval, *BMI* Body mass index, *CHD* Coronary heart disease, *TNM* Tumor nodes metastasis

### OS/ DFS in different TNM stages

**Fig. 2:**
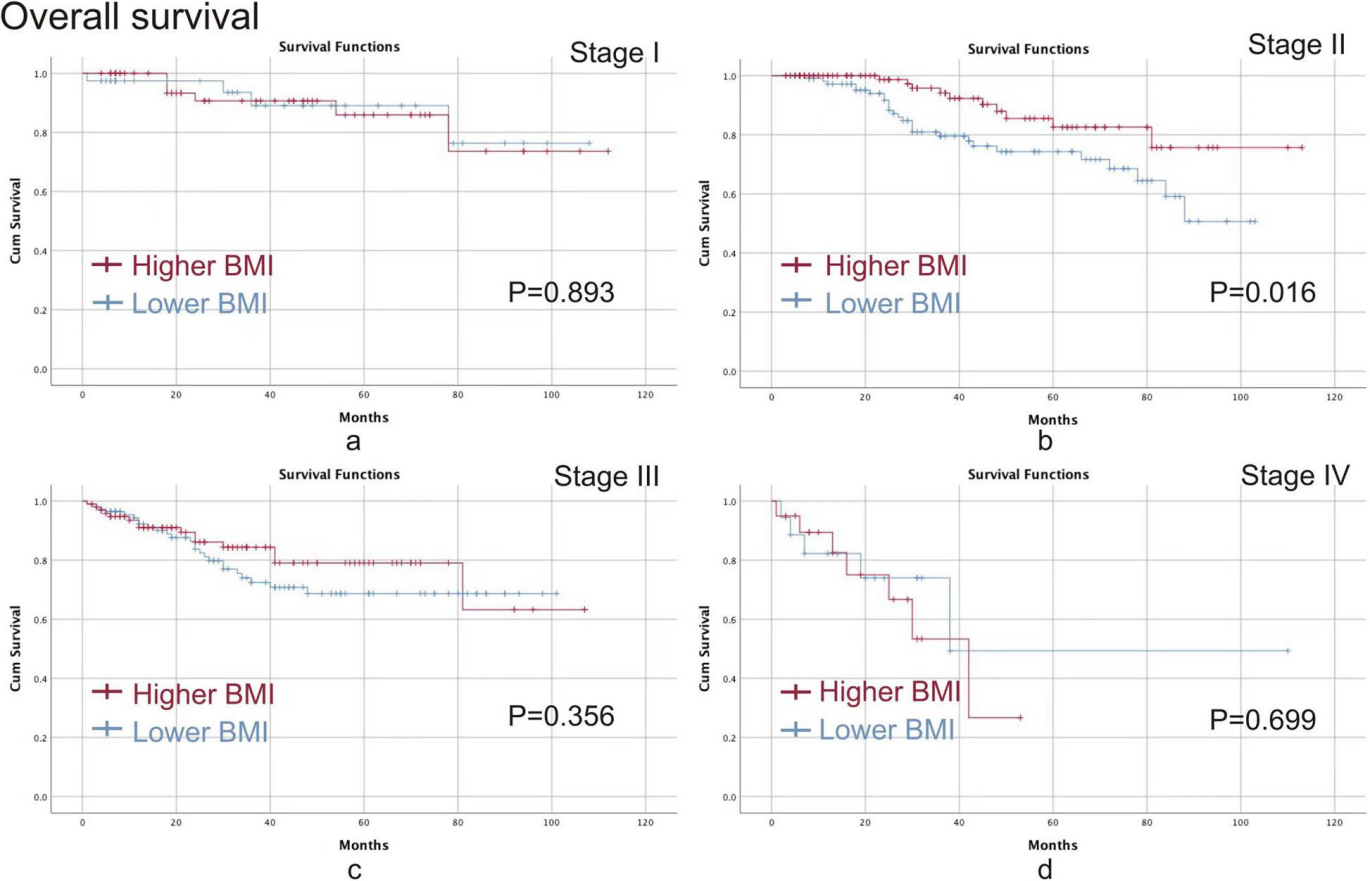
OS between the higher BMI group and the lower BMI group. **a** stage I; **b** stage II; **c** stage III; **d** stage IV. Note: OS, overall survival; BMI, body mass index

**Fig. 3:**
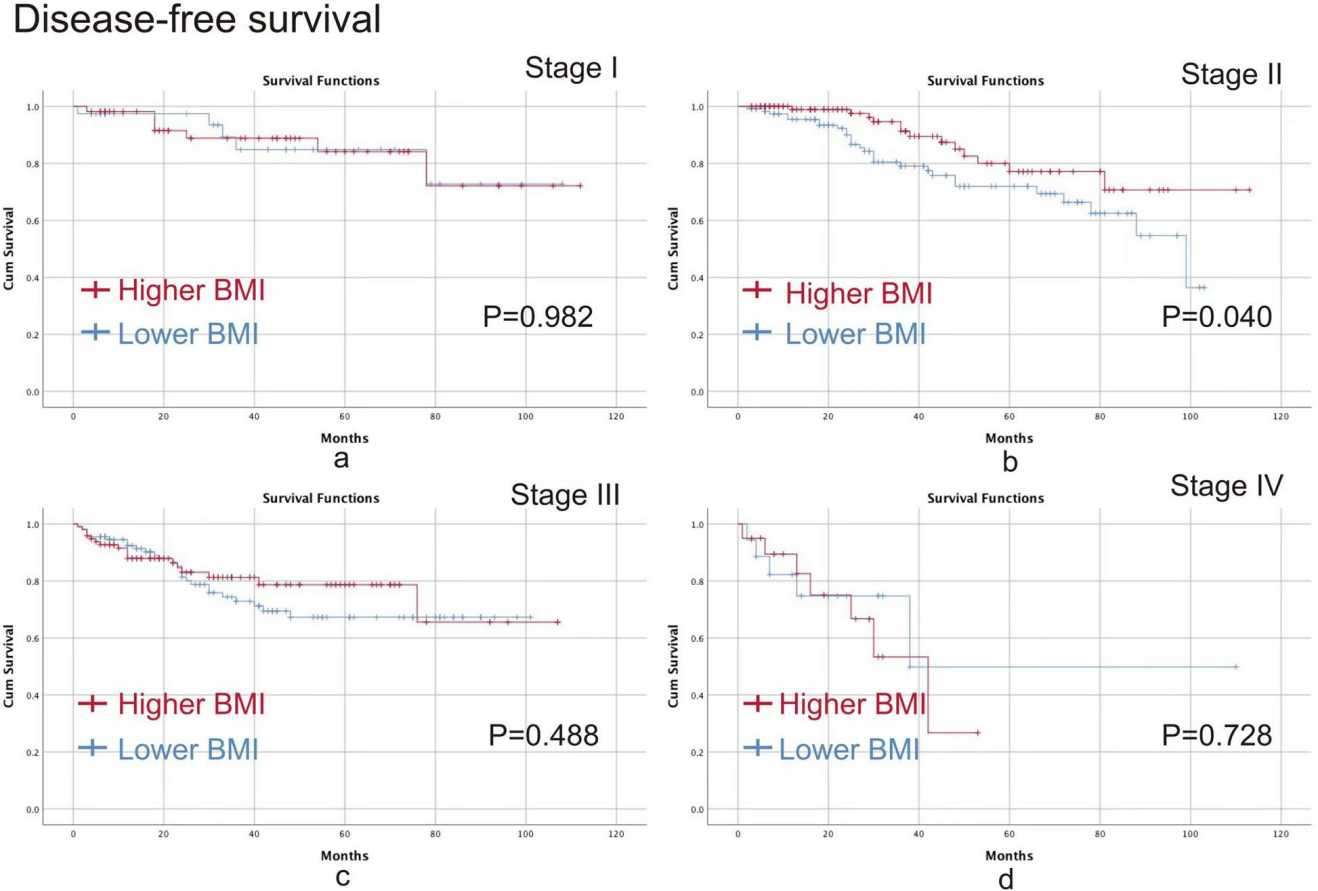
DFS between the higher BMI group and the lower BMI group. **a** stage I; **b**, stage II; **c** stage III; **d** stage IV. Note: DFS, disease-free survival; BMI, body mass index

We conducted Kaplan–Meier curve to analyze the specific effect of BMI on different TNM stages. The higher BMI group had better OS (*p* = 0.016) and DFS (*p* = 0.040) than the lower BMI group in terms of stage II CRC, however, no significant difference was found in other TNM stages in terms of OS and DFS (*p* > 0.05) (Figs. 2 and  3).

Therefore, we conducted univariate and multivariate analysis of OS/ DFS of stage II CRC. As for OS, age (*p* = 0.025, HR = 2.392, 95% CI = 1.113–5.140), BMI (*p* = 0.019, HR = 0.413, 95% CI = 0.197–0.864) and major complications (*p* = 0.046, HR = 3.461, 95% CI = 1.025–11.686) were independent factors of stage II CRC. Interaction analysis of age and BMI revealed no significant difference (*p* = 0.501 > 0.05) (Table 5). In terms of DFS, age (*p* = 0.035, HR = 2.069, 95% CI = 1.053–4.066) was an independent prognostic factor of stage II CRC. Interaction analysis of age and BMI revealed no significant difference (*p* = 0.934 > 0.05). (Table 6).

**Table 5:** Univariate and multivariate analysis of overall survival of stage II CRC patients

Risk factors	Univariate analysis		Multivariate analysis	
	HR (95% CI)	*P* value	HR (95% CI)	*P* value
Age (> / ≤ 68, years)	2.639 (1.238–5.622)	0.012*	2.392 (1.113–5.140)	0.025*
Sex (female/male)	0.947 (0.472–1.896)	0.877		
BMI (> / ≤ 23.4 kg/m^2^)	0.418 (0.202–0.868)	0.019*	0.413 (0.197–0.864)	0.019*
Hypertension (yes/no)	0.980 (0.659–1.459)	0.922		
Tumor site (colon/ rectum)	1.439 (0.719–2.882)	0.304		
Vessel invasion (yes/no)	1.728 (0.235–12.712)	0.591		
Adjuvant therapy (yes/no)	1.058 (0.144–7.798)	0.956		
Smoking (yes/no)	1.081 (0.556–2.099)	0.819		
Drinking (yes/no)	1.442 (0.728–2.857)	0.293		
Family history (yes/no)	2.064 (0.632–6.746)	0.230		
CHD (yes/no)	1.503 (0.526–4.294)	0.447		
Overall complications (yes/no)	1.642 (0.849–3.175)	0.141		
Major complications (yes/no)	3.381 (1.025–11.149)	0.045*	3.461 (1.025–11.686)	0.046*

Note: *P-value < 0.05

*Abbreviations*: *CRC* Colorectal cancer, *HR* Hazard ratio, *CI* Confidence interval, *BMI* Body mass index, *CHD* Coronary heart disease, *TNM* Tumor nodes metastasis

**Table 6:** Univariate and multivariate analysis of disease-free survival of stage II CRC patients

Risk factors	Univariate analysis		Multivariate analysis	
	HR (95% CI)	*P* value	HR (95% CI)	*P* value
Age (> / ≤ 68, years)	2.119 (1.080–4.159)	0.029*	2.069 (1.053–4.066)	0.035*
Sex (female/male)	1.196 (0.638–2.244)	0.577		
BMI (> / ≤ 23.4 kg/m^2^)	0.509 (0.264–0.984)	0.045*	0.523 (0.270–1.012)	0.054
Hypertension (yes/no)	1.110 (0.600–2.054)	0.739		
Tumor site (colon/ rectum)	1.401 (0.733–2.676)	0.308		
Vessel invasion (yes/no)	1.318 (0.180–9.658)	0.786		
Adjuvant therapy (yes/no)	1.782 (0.425–7.473)	0.429		
Smoking (yes/no)	0.859 (0.454–1.625)	0.641		
Drinking (yes/no)	1.442 (0.728–2.857)	0.293		
Family history (yes/no)	1.814 (0.558–5.895)	0.322		
CHD (yes/no)	1.503 (0.526–4.294)	0.720		
Overall complications (yes/no)	1.486 (0.797–2.771)	0.213		
Major complications (yes/no)	2.951 (0.903–9.646)	0.073		

*Note*: *P-value < 0.05

*Abbreviations*: *CRC* Colorectal cancer, *HR* Hazard ratio, *CI* Confidence interval, *BMI* Body mass index, *CHD* Coronary heart disease, *TNM* tumor nodes metastasis

## Discussion

A total of 574 patients with concurrent CRC and T2DM were included in this study. The higher BMI group had higher portion of hypertension and CHD, however, there were no difference of other perioperative information. Furthermore, the higher BMI group had better OS and DFS than the lower BMI group in stage II CRC, however, no significant difference was found in other TNM stages in terms of OS or DFS. Age, TNM stage, overall complications and major complications were independent factors of OS and DFS. Moreover, BMI was an independent factor of OS in stage II CRC.

Higher BMI could increase the risk of CRC [18, 19], and BMI might have an impact on the outcomes and prognosis of CRC surgery [20]. BMI was also related to T2DM and metabolic diseases [21, 22]. Therefore, it is necessary to analyze the exact effect of BMI on diabetic patients with CRC.

However, there were no studies reporting the association of BMI with diabetic CRC patients. Only one study reported the effect of BMI on diabetic patients with gastric cancer [14]. To our knowledge, this is the first study to report the effect of BMI on diabetic patients with CRC.

In this study, we found that the ratio of hypertension and CHD was higher in the higher BMI group compared with the lower BMI group. The probable reason was that BMI was associated with metabolic and cardiovascular diseases [21, 22]. However, there was no difference between the two groups in terms of surgical outcomes. Kwak HD et al. [10] reported that obese patients would cause less lymph nodes harvesting and more blood loss. However, another studies reported that there was no difference in surgical outcomes which was consistent with our study [23, 24]. More studies are needed to analyze the effect of BMI on the surgical outcomes in the future.

The OS and DFS of CRC were affected by some factors including the TNM stage, postoperative complications and age [25–28]. In this study, we found similar independent factors of OS and DFS.

Although BMI was not an independent factor of OS or DFS, it was found statistically different in univariate analysis. Therefore, we hypnotized that BMI might have potential effects on different TNM stages. Therefore, we analyzed the impact of BMI on different TNM stages. It was found that the higher BMI was associated with better OS and DFS in stage II CRC patients. The mechanism was unclear, and few studies had reported the impact of BMI on different TNM stages. Shahjehan F et al. [29] reported that higher BMI had better OS in stage III and IV CRC patients, and another study reported that higher BMI increased the recurrence rate of stage III CRC patients [30]. The possible reason in this study was that higher BMI patients might have more muscle and fat mass, allowing them to cope with the metabolic demands of tumor progression and treatment [31, 32]. Other studies reported lower BMI was associated with cancer-related cachexia and underlying biology in late stage disease patients, which might cause worse prognosis in lower BMI CRC patients [29]. Among patients with higher BMI and CRC, the survival benefit in higher BMI patients might be related to better nutritional status, more optimized drug therapy, more prominent endothelial progenitor cells, lower thromboxane production, higher ghrelin sensitivity and lower TNF-α [33].

Some limitations were existed in this study. First, this was a single-center retrospective study with a relatively short follow-up time; Second, the number of included diabetic patients in stage I and stage II were small, which might result in selection bias; Third, the clinical course and severity of T2DM were not included, which needed to be analyzed in the future. Fourth, pathological factors (lymphatic l invasion, budding, desmoplastic reaction) were lacking in this study. Therefore, larger sample size and multi-center studies with more detailed patients’ information should be conducted in the following experiments.

In conclusion, higher BMI was associated with better OS in diabetic patients with stage II CRC.

## Data Availability

The datasets generated and/or analysed during the current study are not publicly available due [The database from our clinical center were relatively private] but are available from the corresponding author on reasonable request.
